# Supplementary material for: A taxonomic review of the Late Jurassic eucryptodiran turtles from the Jura Mountains (Switzerland and France)
Source: PeerJ. 2014 May 13;2:e369. doi: 10.7717/peerj.369 (PMC4034614; doi:10.7717/peerj.369)
Supplement: Table S1 — Note that not all of these specimens are mentioned in the text, but all have been scrutinized as part of the present study. Note the new specimen numbers for the specimens housed in the Naturmuseum Solothurn (NMS). [file peerj-02-369-s001.docx]

| Specimens | New specimen numbers (NMS) | Classification in Bräm (1965) | This study | Remarks |
| --- | --- | --- | --- | --- |
| MAJ 2005-11-11 | – | *P. etalloni* | *P. etalloni* | Holotype of *P. etalloni* |
| MH 435 | – | *P. etalloni* | *P. etalloni* | Skull-shell association |
| MH So. 563 | – | *–* | *P. etalloni* |  |
| MHNN FOS 977 | – | *P. jaccardi* | *C. jaccardi* | Holotype of *C. jaccardi* |
| MJSN VTT006-52 | – | *–* | *Tr. langii* |  |
| MJSN VTT006-176 | – | *–* | *Tr. langii* |  |
| MJSN VTT006-253 | – | *–* | *Tr. langii* |  |
| MJSN VTT006-290 | – | *–* | *Tr. langii* |  |
| MJSN VTT006-563 | – | *–* | *Tr. langii* |  |
| NMS 1 | NMS 8595 to 8609 | *Th. hugii* | *Th. hugii* | Lectotype of *Th. hugii* |
| NMS 5 | NMS 8612 to 8627 | *E. ignoratum* | *Th. hugii* | Holotype of *E. ignoratum* |
| NMS 9 | NMS 8631 to 8643 | *Th. hugii* | *Th. hugii* |  |
| NMS 12 | NMS 8555 | *Th. hugii* | *Th. hugii* | Holotype of *Th. gresslyi* |
| NMS 15 | NMS 8648 | *Tr. langii* | *Tr. langii* |  |
| NMS 16 | NMS 8554 | *Tr. langii* | *Tr. langii* | Lectotype of *Tr. langii* |
| NMS 20 | NMS 8652 | *Th. hugii* | *Th. hugii* |  |
| NMS 32 | NMS 8556 | *Tr. langii* | *Tr. langii* | Syntype of *Tr. expansa* |
| NMS 33 | NMS 8553 | *Tr. langii* | *Tr. langii* | Syntype of *Tr. expansa* |
| NMS 38 | NMS 8665 | *Tr. langii* | *Tr. langii* | Holotype of *Tr. gibba* |
| NMS 38a | NMS 8666 | *Tr. langii* | *Tr. langii* |  |
| NMS 59 | NMS 8693 | *P. solodurensis* | *P. etalloni* | Lectotype of *P. solodurensis* |
| NMS 60 | NMS 8550 | *P. solodurensis* | *P. etalloni* |  |
| NMS 61 | NMS 8547 | indeterminate | indeterminate |  |
| NMS 62 | NMS 8694 | *Th. moseri* | *'Th.' moseri* |  |
| NMS 64 | NMS 8697 | *Th. moseri* | *'Th.' moseri* |  |
| NMS 66 | NMS 8524 | *P. solodurensis* | *P. etalloni* |  |
| NMS 78 | NMS 8516 | *P. solodurensis* | *P. etalloni* |  |
| NMS 79 | NMS 8517 | *P. solodurensis* | *P. etalloni* |  |
| NMS 81 | NMS 8542 | indeterminate | *P. etalloni* |  |
| NMS 94 | NMS 8533 | *P. solodurensis* | *P. etalloni* |  |
| NMS 101 | NMS 8713 to 8718 | *P. jaccardi* | *C. jaccardi* |  |
| NMS 102a | NMS 8587 | *P. jaccardi* | *C. jaccardi* |  |
| NMS 105 | NMS 8521 | *P. jaccardi* | *C. jaccardi* |  |
| NMS 107 | NMS 8731 | *P. etalloni* | *P. etalloni* |  |
| NMS 111 | NMS 8724 | *Th. moseri* | *'Th.' moseri* |  |
| NMS 116 | NMS 8727 | *P. etalloni* | *P. etalloni* |  |
| NMS 118 | NMS 8514 | *P. sanctaeverenae* | *P. etalloni* | Lectotype of *P. sanctaeverenae* |
| NMS 123 | NMS 8515 | *P. solodurensis* | *P. etalloni* | Lectotype of *P. langii* |
| NMS 124 | NMS 8733 | *E. ignoratum* | *Th. hugii* |  |
| NMS 126 | NMS 8511 | *P. solodurensis* | *P. etalloni* |  |
| NMS 129 | NMS 8510 | *C. picteti* | *C. picteti* | Holotype of *C. picteti* |
| NMS 130 | NMS 8507 | *C. picteti* | *C. picteti* | Holotype of *C. crassa* |
| NMS 132 | NMS 8500 | *Tr. langii* | *Tr. langii* | Holotype of *C. plana* |
| NMS 141 | NMS 8745 | indeterminate | *P. etalloni* |  |
| NMS 412 | NMS 8997 | *E. ignoratum* | *Th. hugii* |  |
| NMS 593 | NMS 9144 | *Th. hugii* | *Th. hugii* |  |
| NMS 606 | NMS 9148 | *P. solodurensis* | *P. etalloni* | Juvenile shell |
| NMS 608 | NMS 9149 | *C. picteti* | *C. picteti* |  |
| NMS 609 | NMS 8447 | *P. etalloni* | *P. etalloni* |  |
| NMS 611 | NMS 9150 | *P. solodurensis* | *P. etalloni* |  |
| NMS 612 | NMS 8443 | *P. jaccardi* | *C. jaccardi* |  |
| NMS 614 | NMS 8446 | *P. solodurensis* | *P. etalloni* |  |
| NMS 615 | NMS 8439 | *P. solodurensis* | *P. etalloni* |  |
| NMS 618 | NMS 9151 | *Th. moseri* | *'Th.' moseri* | Holotype of *'Th.' moseri* |
| NMS 620 | NMS 8436 | indeterminate | *P. etalloni* |  |
| NMS 624 | NMS 8434 | *P. solodurensis* | *P. etalloni* |  |
| NMS 629 | NMS 9153 | *P. etalloni* | *P. etalloni* |  |
| NMS 634 | NMS 8425 | indeterminate | *P. etalloni* |  |
| NMS 669 | NMS 9173 | *P. solodurensis* | *P. etalloni* |  |
| NMS 673 | NMS 9174 | *P. jaccardi* | *C. jaccardi* |  |
| NMS 675 | NMS 8579 | *P. solodurensis* | *P. etalloni* |  |
| NMS 676 | NMS 8578 | *P. etalloni* | *P. etalloni* |  |
| NMS 20981 | (same) | *–* | *Th. hugii* |  |
| NMS 22325 | (same) | *–* | *Th. hugii* |  |
| NMS 22326 | (same) | *–* | *Th. hugii* |  |
| NMS 22327 | (same) | *–* | *Th. hugii* |  |
| NMS 37251 | (same) | *–* | *Th. hugii* | *E. ignoratum* in NMS cat. |
| PMZH A/III 514 | – | *–* | *'Th.' moseri* | Based on Rieppel (1980) |
